# Gene Vector Analysis (Geneva): A unified method to detect differentially-regulated gene sets and similar microarray experiments

**DOI:** 10.1186/1471-2105-9-348

**Published:** 2008-08-22

**Authors:** Stephen W Tanner, Pankaj Agarwal

**Affiliations:** 1 Bioinformatics program, University of California, San Diego, La Jolla, CA 92093-0419, USA; 2 Computational Biology, GlaxoSmithKline Pharmaceuticals R&D, 709 Swedeland Road, UW2230, King of Prussia, PA 19406-0939, USA

## Abstract

**Background:**

Microarray experiments measure changes in the expression of thousands of genes. The resulting lists of genes with changes in expression are then searched for biologically related sets using several divergent methods such as the Fisher Exact Test (as used in multiple GO enrichment tools), Parametric Analysis of Gene Expression (PAGE), Gene Set Enrichment Analysis (GSEA), and the connectivity map.

**Results:**

We describe an analytical method (Geneva: Gene Vector Analysis) to relate genes to biological properties and to other similar experiments in a uniform way. This new method works on both gene sets and on gene lists/vectors as input queries, and can effectively query databases consisting of sets of biologically related sets, or of results from other microarray experiments. We also present an improvement to the null model estimate by using the empirical background distribution drawn from previous experiments. We validated Geneva by rediscovering a number of previous findings, and by finding significant relationships within microarrays in the GEO repository.

**Conclusion:**

Provided a reasonable corpus of previous experiments is available, this method is more accurate than the class label permutation model, especially for data sets with limited number of replicates. Geneva is, moreover, computationally faster because the background distributions can be precomputed. We also provide a standard evaluation data set based on 5 pairs of related experiments that should share similar functional relationships and 28 pairs of unrelated experiments from GEO. Discovering relationships amongst GEO data sets has implications for drug repositioning, and understanding relationships between diseases and drugs.

## Background

High-throughput experiments such as microarrays compare the expression levels of thousands of genes at once. Individual gene readings, when compared to a control, measure the degree to which the gene is up- or down-regulated. Microarray experiments contain significant noise, and typically only a few genes are found whose expression is significantly changed. Recently, several groups have begun to examine microarray experiments from the perspective of biologically related gene sets. There are a large number of such methods based on thresholding the initial microarray results by fold-change or p-value and then using a Fisher exact test to determine significance (e.g. [1]). We use the term *gene set database query *to describe the comparison of a microarray experiment against a database of sets of genes (often with associated biology). The results from a gene set database query consist of biologically meaningful groups, such as the set of all genes annotated with a GO term or a pathway, rather than individual genes. Non-parametric algorithms such as GSEA [2] and PAGE [3] are also available for this type of search. General tools to assist biologists with the analysis are being developed [4,5], and in this context, quantifying the success of particular algorithms becomes even more important.

In addition to queries against a database of biologically meaningful sets, we may wish to query a database consisting of other experiments. We use the term *gene vector database query *to describe a query against a database whose entries are themselves vectors of gene readings. Such a query may aim to find related experiments – for example, queries of signatures against the Connectivity Map corpus were able to identify compounds with similar effects [6].

The naive null model for a gene set database query (in both GSEA and PAGE) is that the genes in the set are drawn independently from the overall distribution. However, many gene sets of biological interest consist of co-regulated genes. The expression responses of these genes will typically be highly correlated. This tight correlation may cause us to reject the naive null model with high confidence, even in cases where the genes are not differentially regulated. One way to compensate for this interdependence of genes within a set is through permutation of class labels [7]. However, a disadvantage of permutation testing is that it requires a large number of replicates. (Note that for experiments involving fewer than 13 microarrays, fewer than 1,000 distinct permutations exist, thus permutation based p-values may be limited to > 0.001). We show that it is more effective to calibrate p-values for each set (or vector) in the database using a large corpus of experiments. Once this calibration is performed, queries can be performed with higher accuracy than permutation tests, and with less computational cost.

Statistical methods which apply to set queries, such as Fisher Exact, may not apply to vector queries. If different statistical methods are used, the p-values from gene set database queries and gene vector database queries may not be comparable. For this reason, we developed a method that can query both sets and vectors, using a common statistical framework. With this method, one can query microarray readings against a database of sets (as in GSEA), or query gene signatures against a database of vectors (as used in the Connectivity Map). In addition, one can use our method to query microarray readings against a database of previous microarray experiments (e.g. to find drugs which offset the transcriptional changes associated with a disease). The source code implementing our query tool, Geneva, is available .

We report the results of evaluation experiments using publicly available microarray experiments from the GEO data repository. Related microarray experiments are those that differ only by (for example) severity of disease, dose of compound, or sampling of subjects. Gene set enrichment methods should identify very similar enriched sets for related experiments. We formalize this idea to identify 5 pairs of related experiments from GEO as an evaluation set, thus, extending the data sets from PAGE [3]. We also use 28 pairs of mismatched (or unrelated) experiments as a negative set. This provides an objective framework to evaluate multiple methods as to their accuracy. The value of standard evaluation data sets is well proven – for example, see the influence of the Burset and Guigo data set in gene-finding [8]. Using this evaluation data enables us to compare statistical measures within Geneva.

We divide the generalized gene vector analysis problem into three steps. The first step is the acquisition of a *reading *for each gene to be used for querying, and compiling a database of gene sets and/or gene vectors. The second step is the calculation of an *enrichment score *for each gene set (or gene vector) in the database. The third step is the conversion of these enrichment scores into *p-values*, using the distribution of enrichment scores on a corpus of data. The computation of these values is described in the Methods.

## Results

**Figure 1:**
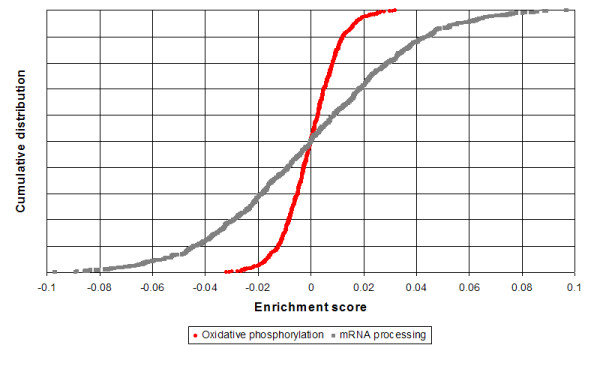
**Cumulative distribution functions of Pearson enrichment scores for two gene sets across the CMAP corpus.** There are clear differences in the variance of these two distributions of two gene sets. However, the empirical distribution of scores across the corpus fits a normal distribution well for most gene sets. For each gene set, we calculated a p-value based on the specific normal distribution associated with that gene set.

The distribution of enrichment scores for a given gene set across the corpus reflects the co-regulation (formally: the correlation in transcription changes) of the genes across various treatments. Figure 1 compares these distributions for two gene sets: A set of 118 genes related to oxidative phosphorylation, and a large set of 1,222 genes related to mRNA processing. For an example of how p-value calibration provides improved query results, let us consider a data-set (GDS287) comparing muscle tissue from young and aged males. Using a naive query that performs no p-value calibration, we obtained a p-value of 1.2 × 10^-34 ^for the mRNA processing set, much lower than the value for oxidative phosphorylation (6.4 × 10^-11^). Similar results were observed using PAGE [3]. However, after calibration against the CMAP corpus (see Methods), this ordering is reversed, and the p-value for mRNA processing is no longer significant after correcting for multiple hypothesis testing. Naive queries frequently detect the mRNA processing set as enriched – indeed; it receives an uncorrected p-value below 0.05 in the *majority *of the 463 CMAP experiments. This demonstrates that the naive null hypothesis does not suffice to filter out false positives based on gene sets comprised of highly co-regulated genes from gene set queries.

**Figure 2:**
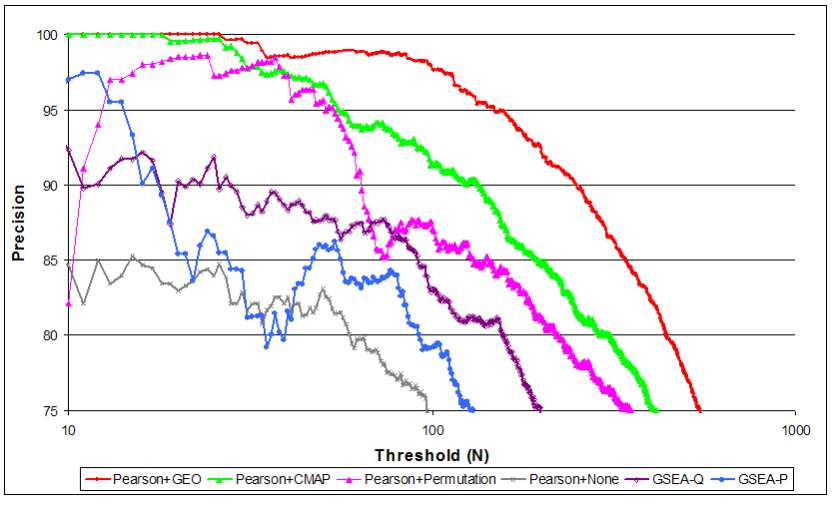
**Precision (1-FDR) of gene set queries methods with Pearson based p-values calibrated on GEO and CMAP compared to permutation based p-values and no permutation.** Also included, for comparison are GSEA q-value (based on FDR) and GSEA p-value (based on FWER). We compared Precision across the various queries for threshold of gene sets (N) plotted on the x-axis (as described in Methods).

As described in the Methods, we computed the false discovery rate for queries across pairs of related and unrelated experiments (Figure 2). If p-value calibration is performed, many more gene sets are observed at a 10% false discovery rate. The results also demonstrate that either large corpus provides a reasonable training set for p-value calibration, as queries calibrated with either GEO or CMAP perform significantly better than those without calibration. The GEO corpus has the advantage that it includes a wide array of treatments and tissue types, and that it uses t-scores (available only for the GEO corpus) rather than fold changes (available for the CMAP corpus). On the other hand, the CMAP corpus is somewhat larger, and has the advantage that it was generated by one lab with high reproducibility. The GEO corpus was arguably more effective, as measured by the slower decrease in precision. However, when we list the top 10 gene sets for these experiments (as measured by product of p-values), the lists reported using calibration against the CMAP corpus appeared to be most biologically reasonable (in our subjective opinion).

Calibrating p-values using a corpus of experiments is less expensive computationally than using a permutation of class labels, particularly if many queries will be run against the same database of gene sets. The initial corpus calibration is time-consuming (requiring approximately 1 day of running time on a typical desktop PC), but need only be done once for each gene set. Perhaps surprisingly, our results show that calibrating p-values across a corpus of experiments yielded higher accuracy than generating p-values by permuting the class labels. However, we note that permutation of class labels is clearly more effective than no p-value calibration at all.

**Figure 3:**
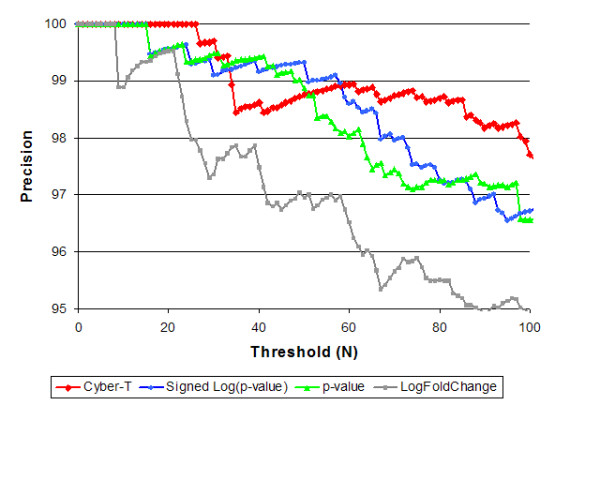
**Comparison of query accuracy, on the evaluation set, with p-values calibrated against the GEO corpus using Pearson correlation.** Queries based upon signed p-value were more effective than just p-value. Cyber-T was also extremely effective especially for N > 60. Using log fold changes as gene values was least effective consistently, perhaps due to the noise in the log fold change for genes with low expression.

In a related experiment, we compared the query precision obtained when using the Cyber-T statistic, Cyber-T p-values, or log fold change as our gene readings (Figure 3). Queries using the Cyber-T statistic or p-value are noticeably more accurate than those driven solely by log fold change. This reflects the large amount of noise in fold-change measurements for genes expressed at a low level. Not surprisingly, precision declines as the significance threshold drops (i.e. N increases).

**Figure 4:**
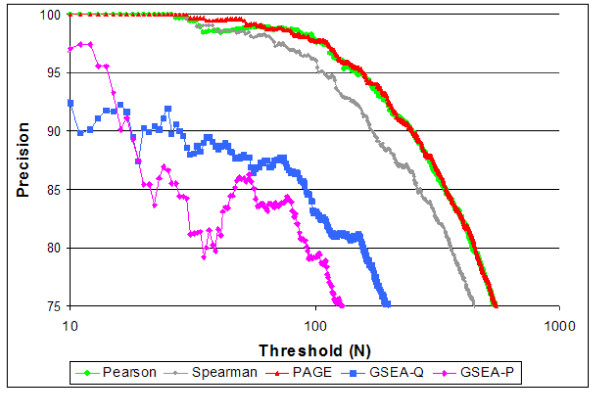
**Comparison of query accuracy, for the evaluation set, using various enrichment models.** This is based on the GEO corpus using the Cyber-T. PAGE produces the best results. Pearson is a very close second; an additional advantage of Pearson correlation is that it is effective for queries against vectors and gene sets.

A final evaluation experiment compared the accuracy obtained using several different enrichment models (Figure 4). Pearson correlation is more accurate than Spearman correlation, as might be expected when comparing parametric and non-parametric models. However, Pearson and PAGE were almost identical. Noticeably, both the FDR q-value and FWER p-values from GSEA performed worse, possibly because we did not calibrate those p-values.

### Gene set results

Table 1 lists several of the top gene sets returned for the five pairs of experiments described in Methods. (A full table of gene sets, together with the corresponding Affymetrix probe IDs, is available in Additional file 1). Some gene sets of clear biological interest arise. For example, the set of genes annotated with the biological process "Long-term memory" was down-regulated in the Alzheimer's disease samples. Gene sets related to immune response were differentially regulated in response to malaria infection. As reported previously [3], gene sets related to glycolysis and the TCA cycle are differentially regulated in young and aged muscle.

**Table 1:** Top-scoring differentially expressed gene sets found for pairs of related microarray experiments (from the category in column 1 above) using Geneva.

**Experiment**	**Rank**	**p-value**	**Name**	**Source**
Muscle	1	7.89E-10	Glycolysis_and_Gluconeogenesis	GenMAPP
Muscle	2	6.93E-09	Costamere: CC	GOA
Muscle	3	4.37E-07	superpathway of glycolysis, pyruvate dehydrogenase, TCA, and glyoxylate bypass	HumanCyc
Muscle	4	4.86E-07	Contractile Fiber Part: CC	GOA
Muscle	5	6.54E-07	Z Disc: CC	GOA
Muscle	6	9.20E-07	Small Leucine-Rich Proteoglycan (SLRP) Molecules	BioCarta
Muscle	7	1.69E-06	aspartate degradation II	HumanCyc
Muscle	8	4.80E-06	Myofibril: CC	GOA
Muscle	9	4.87E-06	gluconeogenesis	HumanCyc
Muscle	10	5.38E-06	Contractile Fiber: CC	GOA
Malaria	1	1.60E-08	Immune Response-Regulating Signal Transduction: BP	GOA
Malaria	2	1.60E-08	Immune Response-Regulating Cell Surface Receptor Signaling Pathway: BP	GOA
Malaria	3	1.60E-08	Immune Response-Activating Signal Transduction: BP	GOA
Malaria	4	1.60E-08	Immune Response-Activating Cell Surface Receptor Signaling Pathway: BP	GOA
Malaria	5	1.60E-08	Antigen Receptor-Mediated Signaling Pathway: BP	GOA
Malaria	6	1.67E-08	T Cell Receptor Signaling Pathway: BP	GOA
Malaria	7	1.76E-08	Regulation Of T Cell Receptor Signaling Pathway: BP	GOA
Malaria	8	2.69E-08	Regulation Of Antigen Receptor-Mediated Signaling Pathway: BP	GOA
Malaria	9	2.64E-07	Activation Of Csk By cAMP-Dependent Protein Kinase Inhibits Signaling Through The T Cell Receptor	BioCarta
Malaria	10	5.02E-07	Locomotion: BP	GOA
AD	1	1.13E-11	Proton-Transporting Two-Sector ATPase Complex: CC	GOA
AD	2	1.13E-11	Hydrogen-Translocating V-Type ATPase Complex: CC	GOA
AD	3	9.31E-11	Long-Term Memory: BP	GOA
AD	4	4.91E-10	aspartate degradation II	HumanCyc
AD	5	1.78E-09	Proton-Transporting ATP Synthase Complex: CC	GOA
AD	6	1.78E-09	Proton-Transporting ATP Synthase Complex (sensu Eukaryota): CC	GOA
AD	7	1.78E-09	Hydrogen-Translocating F-Type ATPase Complex: CC	GOA
AD	8	1.95E-09	Hydrogen Ion Transporter Activity: MF	GOA
AD	9	6.44E-09	Monovalent Inorganic Cation Transporter Activity: MF	GOA
AD	10	7.75E-09	Ubiquinol-Cytochrome-C Reductase Activity: MF	GOA

Pearson correlation was used, and was calibrated against a corpus of experiments from GEO (see Methods).

The p-value reported is the product of the p-values for the two related experiments.

The original study of the Alzheimer's disease samples [9] identified several differentially expressed gene sets using a modified Fisher's exact test. Several biological processes were identified again by our study, including downregulation of ATP biosynthesis and GPCR signaling, and upregulation of apoptosis. We believe that the reliability of our results is improved by the use of a parametric statistic, as well as a more reasonable null model, which accounts for co-regulated gene sets.

### Vector query results

As described in Methods, we computed the correlation of the Cyber-T vectors for all pairs of experiments in the GEO corpus. Given these values, we performed vector queries, to identify all experiments significantly related to a microarray experiment. Such experiments may affect the cell similarly (e.g. exposure to related compounds), or may perturb similar pathways with opposite effects (e.g. disease response versus exposure to a treatment). This query involved approximately 800,000 pairwise comparisons, and required 3 CPU days of running time on a compute-cluster. We examined the query results up to a p-value of 0.05.

We examined the query results for pairs of related experiments (see Methods). As expected, the two muscle data-sets (GDS287 and GDS472) are related to each other (p-value 3.55 × 10^-6^). Two other data-sets were significantly similar – a study of sarcopenia (GDS749) and a study of the effects of exercise on muscle in elderly males (GDS1340). These results show the effectiveness of exercise in offsetting age-associated muscle loss at the transcriptional level. The Alzheimer's disease experiment (GDS810) was similar to an experiment on bipolar disorder (GDS2190), suggesting these disorders might be similar.

Our full GEO against GEO results are reported [see Additional file 2]. Relationships between compounds can be discovered by this kind of undirected data-mining. For instance, a close relationship was observed between experiments exposing a prostate cancer cell line to two different androgens: DHT (GDS2057) and methyltrienolone/R1881 (GDS536). Hits were also seen for experiments with the transcriptional changes induced by the estrogen hormone estradiol (GDS1549) and by the estrogen receptor agonist tamoxifen (GDS2367).

Other hits come from experiments with related treatments – comparisons of transcription in blood versus liver (GDS1023) and kidney versus liver (GDS1663) were closely related, presumably due to transcription of liver-specific genes. Some of the confident query hits come from experiments from different labs which applied essentially the same treatments – for instance GDS1549 and GDS2367 both measure the effects of estradiol on breast cancer cell lines. In the future, programmatically examining meta-data (e.g. from MIAME) may permit highlighting the most interesting search hits by filtering out closely related experiments.

## Discussion and conclusion

The high-throughput gene collection database query problem can be formulated in several ways, focusing on either gene set database queries or gene vector database queries. The use of a parametric statistical framework that accepts either gene names or gene values as input is important, particularly when combining heterogeneous data-sources (e.g. microarrays and literature-curated gene lists). Queries against both sets and vectors work well with simple metrics such as Pearson correlation, provided that p-values are calibrated properly.

Calibration of gene set database queries against a corpus of experiments provides much more accurate results than using a naive null model. Calibration against a training corpus is certainly not ideal for all situations. In cases where a suitable corpus is not available (e.g. if one is investigating an organism that has not yet been extensively studied), class label permutation is the only practical approach. If a set of genes is not significantly expressed in the training corpus, then the training corpus will not adequately measure their degree of correlation. Therefore, it is desirable to use a training corpus containing as wide a variety of tissues and conditions as possible.

Identifying standard data sets that can be used to compare different algorithms and different metric is beneficial. Our proposed standard data set of 5 related pairs of experiments and 28 unrelated pairs of experiments is an advance over evaluations based on one or two anecdotal comparisons. However, there is much room for improvement by extending the numbers of both related and unrelated pairs and further reducing the bias caused by any single experiment. We also recognize that the figures do not provide statistical tests to determine if in fact a method is superior to another. Again this was impossible given the small size of the data set. But it also cautions us to not over interpret the findings in figures 2 and 4.

The emergence of large microarray repositories, such as GEO, provide researchers with the ability to search for experiments with similar (or opposite) gene changes. Such searches provide an ideal approach to find compounds which offset the gene expression changes associated with disease states. Calibration of p-values using a corpus of experiments significantly improves the accuracy of such queries by providing a reasonable null model without the need for large numbers of controls.

## Methods

### Gene Readings

In order to perform a query, we need a single reading quantifying the degree of up- or down-regulation of each gene. The gene readings for the genes included in the microarray will be represented as a query vector of length N, whose n^th ^value represents the change in transcription of the n^th ^gene. The levels of transcription of each gene in the public data-sets we used were initially quantified using MAS5 [10] or related methods. To quantify the up- or down-regulation of each gene, we employed the Cyber-T algorithm [11]. The Cyber-T statistic itself is retained as the reading for a gene. The Cyber-T statistic has the advantage that it reflects both direction (up-versus down-regulation) and confidence. A variety of methods are available to quantify up- and down-regulation [12], which can be incorporated similarly. In addition, we tried applying log fold change (which reflects direction) and the Cyber-T p-value (which reflects only confidence). In addition, p-values from SAM [13] were computed and tested, and found to give similar results to Cyber-T p-values (data not shown).

The Connectivity Map (CMAP) corpus consists of a total of 463 microarray experiments involving the exposure of human cell cultures to various perturbagens [6]. As a second corpus, we obtained all GEO data-sets available for the Affymetrix HG-U133A chip (GPL96) as of February 1^st^, 2007. The SOFT-format files for each data-set were parsed, and expression differences were measured using Cyber-T for each pair of sample sets which (a) contained three or more entries per set, and (b) were disjoint. To avoid over-representing particular treatments in our corpus, we selected at most three such comparisons per data-set. The resulting corpus contains a total of 285 gene vectors. For each comparison (A vs. B), we also added the reverse comparison (B vs. A) which increased the number of corpus to 570 vectors. This was done for technical and expository reasons as it made the distributions of scores symmetrical.

A database of gene sets was constructed from several sources: GOA [14], GenMAPP [15], HumanCyc [16], BioCarta , and TRANSFAC [17]. Gene identifiers from the source databases, along with Affymetrix microarrays, are mapped to a collection of common identifiers. Because small gene sets do not lead to statistically significant results, we ignored any set containing fewer than five genes. A total of 4,256 gene sets of sufficient size were used.

### Enrichment Scores

Given a query vector of gene readings (as described above) and a gene set, we considered several statistical models for computing an enrichment score for the gene set:

• Pearson correlation. We construct a binary membership vector for the set. This membership vector's n^th ^entry is 1 if the n^th ^gene is a member of the set, and 0 otherwise. We then compute the Pearson correlation between the membership vector and the query vector. The enrichment score is the Pearson correlation coefficient, *r*.

• Spearman correlation. As with Pearson correlation, we first construct a binary membership vector for the set. We then compute a Spearman (rank-based) correlation, *ρ*, between the membership vector and the query vector. The enrichment score is the variable *t*, defined as:

$$t=\frac{\rho \sqrt{N-2}}{\sqrt{1-\rho^{2}}}$$

• PAGE. We implemented the Parametric Analysis of Gene Set Enrichment (PAGE) method as described by Kim & Volsky [5]. PAGE is based on using the normal distribution for statistical inference, and is possibly more sensitive than GSEA.

The two correlation-based methods have the advantage that they apply equally well to queries against a database of vectors. The accuracy of these methods was compared on an evaluation data set.

In the past, researchers have compiled a set of genes of interest from a microarray experiment (e.g. two-fold or greater change in expression), then compared the set against a database of biologically related genes using Fisher's Exact Test. Geneva can be used in essentially the same way if Cyber-T is replaced by a binary-valued vector set to one for precisely those genes of interest. However, querying based upon the readings themselves is more informative than applying an arbitrary cutoff and then querying upon gene sets.

### Calibration of p-values

For the GEO corpus and for each gene set, we fitted a normal distribution to the empirical distribution of the enrichment scores. (See Figure 1 for an empirical cumulative distribution for two different gene sets.) The inferred mean (*μ*) and standard deviation (*σ*) parameters of the normal distribution were then used to compute p-values for that gene set for all queries. This was done independently for each of the three enrichment score methods: Pearson, Spearman, and PAGE, and also done for the CMAP corpus in addition to the GEO corpus.

Under reasonable assumptions, the theoretical distribution of Pearson correlation scores follows a normal distribution whose variance is inversely proportional to the number of genes [18]. In practice, the distribution of Pearson correlation scores for gene sets in our database across the corpus is indeed fit well by a normal distribution, but with a standard deviation that varies between gene sets. The variance of the enrichment score distribution correlates with size (r = 0.41), but is also affected by the degree of co-regulation.

We expect the distribution of the enrichment scores across the corpus to follow a normal distribution. We evaluated the quality of the fit to the normal distribution using the Kolmogorov-Smirnov statistic. (This KS test was used to test for normality, and should not be confused with the use of KS test in GSEA.) The median p-values for the GEO and CMAP corpora were 0.87 and 0.60 respectively. Thus, we could not reject the hypothesis that the p-values are normally distributed. Similarly, Spearman correlation p-values follow a normal distribution (median p-values 0.61 and 0.53), as do Z-scores (p-values 0.88 and 0.68). The standard deviations of enrichment scores for gene sets across the two corpora are tightly correlated (r = 0.87). This suggests that any sufficiently large and diverse corpus provides a reasonable measurement of the degree to which genes in a set are co-regulated.

### Evaluation of query algorithm

We obtained several publicly-accessible microarray data-sets from the GEO repository [19,20]. Five pairs of related experiments were used in our evaluation experiment, as follows:

• Muscle: Muscle tissue from old males (67–75 years) vs. young (21–27 years) males (GDS287) and old females (65–71 years) vs. young (20–29 years) females (GDS472) [21].

• Malaria: Whole blood from children with mild malaria vs. healthy children and severe malaria vs. healthy children (GDS1971)

• AD: Brain tissue from subjects with moderate AD (Alzheimer's disease) vs. normal and with severe AD vs. normal (GDS810) [9].

• Glioma: Grade III gliomas vs. control (non-tumor) cells and grade IV gliomas vs. control cells (GDS1962) [22].

• Obesity: Skeletal muscle tissue samples from obese vs. non-obese and morbidly obese vs. non-obese subjects (GDS268) [23].

The pairs of experiments described above are considered *related*, as they involve similar biological changes and should affect transcription in similar ways. In order to quantify the performance of our queries, we tabulate the gene sets that are considered as significant for both the related experiments. If a gene set is found to be enriched in both experiments, we have increased confidence that the gene set is indeed undergoing a biologically relevant change in expression. By contrast, we expect to see few (if any) shared gene sets between two experiments chosen from different biological conditions. In practice, some overlap was seen between some of those pairs, for instance, we saw some overlap between the effects on muscle tissues of obesity and aging. So we selected an even cleaner negative control set by picking a collection of seven *unrelated *experiment pairs (as close to "biologically disjoint" as possible), which should share few up- or down-regulated genes (Table 2).

**Table 2:** Treatments considered *unrelated *for the purpose of evaluation experiments.

**Pair A**	**Pair B**
Muscle	Malaria
Muscle	Glioma
Malaria	Glioma
Malaria	Obesity
AD	Malaria
AD	Obesity
Glioma	Obesity

Each treatment has two experiments, for a total of 28 unrelated experiment pairs.

We listed the top N gene sets reported as enriched for any of the ten evaluation experiments, for N ranging from 1 to 1000. We then checked if a gene set is reported as enriched for two experiments. We count the number of such shared gene sets for related experiments (e.g. obesity in male and in female), denoting the count as *V*. Similarly, let *I *denote the number of shared gene sets for unrelated experiments; these are (to a first approximation) all invalid. These gene sets shared between unrelated experiments serve as an estimate of the number of spurious gene sets shared between related experiments. For any given N, the false discovery rate (FDR) [24] for gene sets shared between related experiments can be readily computed (for V > I) as *cI/V*. Here the scaling factor, *c *is the number of related experiment pairs divided by the number of unrelated experiment pairs. We can also define Precision to be (*1-FDR*) = *(1-cI/V*). This Precision is plotted against N in figures 3 &4. Method A is considered better than method B for threshold N if it has higher Precision. For each method after evaluation on the above data set, we selected an FDR cutoff of 10% for our comparisons (see Table 1 for Pearson hits at FDR = 10%).

### Queries against a gene vector database

The Pearson and Spearman correlation enrichment models can be applied equally well to queries against a database of vectors. As a test of this procedure, we measured differential expression using Cyber-T for all data sets in the GEO corpus (described above), then performed an all-against-all vector query. We modeled the distribution of correlation values R for a given data-set X with a normal distribution. This enables us to compute the p-value, P_X_(R), for a given value of R. When comparing vectors X and Y, a p-value for the association of X and Y is computed as the geometric mean of P_X_(R) and P_Y_(R). This score reflects the significance of a particular correlation R relative to the correlation values observed for X and Y across the entire corpus. In the absence of a training set of query results, we examined the query results for several GEO data-sets to determine whether they were biologically reasonable.

## Authors' contributions

SWT implemented the algorithm and built the test data-set. PA formulated the problem and directed the comparison of methods. Both authors prepared the final manuscript.

## Supplementary Material

Additional file 1 Enriched gene sets. Click here for file

Additional file 2 GEO vs. GEO query. Click here for file
